# Prenatal stress shapes discrete responses during early recovery from repeated adult stress

**DOI:** 10.3389/fncel.2026.1793298

**Published:** 2026-05-13

**Authors:** Branden G. Verosky, Jessica L. Anderson, Helen J. Chen, Therese A. Rajasekera, Felix Yang, Tamar L. Gur

**Affiliations:** 1 Institute for Brain, Behavior, and Immunity, The Ohio State University Wexner Medical Center, Columbus, OH, United States; 2 Department of Psychiatry and Behavioral Health, The Ohio State University Wexner Medical Center, Columbus, OH, United States; 3 Neuroscience Graduate Program, The Ohio State University, Columbus, OH, United States; 4 The Ohio State University College of Medicine, Columbus, OH, United States; 5 Department of Obstetrics and Gynecology, The Ohio State University Wexner Medical Center, Columbus, OH, United States; 6 Department of Neuroscience, College of Medicine, The Ohio State University, Columbus, OH, United States; 7 Soter Women’s Health Research Program, College of Medicine, The Ohio State University, Columbus, OH, United States

**Keywords:** corticosterone, inflammation, prenatal stress, second hit, stress

## Abstract

**Introduction:**

Prenatal stress is associated with increased risk for psychiatric disorders in offspring, yet many exposed individuals do not develop psychopathology, suggesting prenatal stress may confer a latent vulnerability that emerges only under later-life challenge. We have previously demonstrated long term neuroinflammation and behavioral changes following exposure to prenatal stress, but had not examined whether the offspring were vulnerable to adult stressors, which is translationally relevant.

**Methods:**

To test whether prenatal stress alters the stress response and stress recovery following repeated stress, pregnant dams underwent prenatal restraint stress (GD10.5–GD16.5), and adult offspring of both sexes were assigned to a 1-week adult restraint regimen or adult control in a 2 × 2 factorial design. Anxiety-like and social behavior was assessed after the stress paradigm in adulthood, and endocrine and transcriptional endpoints were measured immediately after stress and during early recovery.

**Results:**

Repeated adult restraint elicited a robust but transient corticosterone response and induced broad, largely time-limited stress-responsive transcription across prefrontal cortex, hippocampus, amygdala, and hypothalamus. Prenatal stress did not globally potentiate HPA-axis output or canonical glucocorticoid-responsive gene induction under this strong adult stressor, but instead produced selective, context-dependent effects on stress- related behavior and region- and sex-specific gene regulation during the acute and recovery phases.

**Discussion:**

Collectively, the data indicate that under a strong repeated adult stressor, prenatal stress is associated with selective differences in the pattern and timing of behavioral and transcriptional responses across stress-regulatory circuitry, rather than a uniform modulation in HPA-axis output. This pattern underscores a complex, long-term interplay between prenatal stress exposures and later-life stressors, which together may shape vulnerability or resilience to adult psychopathology.

## 1 Introduction

Maternal stress during pregnancy is associated with increased risk for psychiatric disorders in offspring (Khashan et al., 2008; Pearson et al., 2013; Manzari et al., 2019), yet many exposed individuals never develop overt psychopathology. This heterogeneity supports the idea that prenatal stress may confer a latent vulnerability that remains phenotypically silent until later-life challenge and may manifest differently across sexes, consistent with the sex-biased prevalence and presentation of many psychiatric disorders. In line with this view, prenatal stress may shape stress-related neural circuitry and HPA-axis reactivity, biasing downstream behavioral and neuroendocrine responses when offspring encounter subsequent stressors.

Clinical studies have associated prenatal maternal stress and psychopathology with differences in offspring stress physiology, most consistently reflected in altered cortisol reactivity rather than baseline cortisol levels. Across infancy and early childhood, prenatal maternal depression has been associated with disrupted basal cortisol (Molenaar et al., 2019; Stonawski et al., 2019) and cortisol reactivity to acute stressors (Tollenaar et al., 2011; Osborne et al., 2018; Swales et al., 2018). While the direction of these associations is mixed, this likely reflects the differences in gestational timing of maternal symptom assessment, child age assessment, and the nature of the acute stressor evaluated. Neuroimaging studies complement these endocrine associations by linking antenatal maternal affective symptoms to altered amygdala development (Buss et al., 2012; Wen et al., 2017; Qiu et al., 2021) and amygdala–prefrontal connectivity (Posner et al., 2016), suggesting potential neural changes for the altered stress responsivity. Together, these data support the hypothesis that prenatal stress exposures can modulate offspring stress responsivity. Given that development of psychiatric disorders and stress-related phenotypes differ across sexes, assessing both males and females is important for determining whether prenatal stress models capture shared versus sex-specific outcomes, thereby strengthening their translational relevance and validity.

The hypothesis that prenatal stress can alter stress responsiveness in a way that produces subtle changes at baseline that becomes evident under later challenge, often termed a “second hit”, is supported in rodent models. Across rodent prenatal stress paradigms, adult offspring demonstrate a heightened corticosterone response after acute stress in a sex- and paradigm-dependent manner (Brunton and Russell, 2010; Enayati et al., 2020) although this potentiated response is not observed with chronic stress (Van den Hove et al., 2014). This altered corticosterone response corresponds with persistent changes within stress-regulatory circuitry, such as reduced glucocorticoid receptor in prefrontal cortex (PFC) and hippocampus (Green et al., 2011) and sex-dependent modulation of stress-evoked PFC *Bdnf* expression (Luoni et al., 2016). Along similar lines, we have previously reported a heightened corticosterone response to social interaction in adult male offspring of prenatally stressed dams (Gur et al., 2019). However, most work has focused on acute challenges and therefore cannot resolve whether prenatal stress primarily alters the response to acute stressors or the recovery of stress signaling following repeated challenge.

In parallel with HPA-axis programming, prenatal stress has also been shown to cause long-term alterations in inflammatory signaling within the offspring brain. Preclinical studies, including our prior work, show that prenatal stress increases inflammatory signaling in the fetal brain and produces persistent, sex-dependent alterations in microglia that persist into adulthood, including changes in density, morphology, pro-inflammatory gene expression, and later immune responsivity (Diz-Chaves et al., 2012; Diz-Chaves et al., 2013; Slusarczyk et al., 2015; Gumusoglu et al., 2017; Gur et al., 2017; Gur et al., 2019; Chen et al., 2020). Maternal stress during pregnancy has also been associated with altered cytokine profiles, including elevated pro-inflammatory cytokines IL-6 and TNF-α and reduced IL-10 (Coussons-Read et al., 2007; Haeri et al., 2013; Osborne et al., 2018), supporting the idea that immune perturbations may contribute to the programming of later stress vulnerability.

To extend our previous studies and determine whether prenatal stress alters feedback control and post-stress normalization of the stress response following repeated stress, we used a 2 × 2 factorial design combining prenatal restraint stress (GD10.5–GD16.5) followed by 1-week of daily restraint stress in the adult offspring of both sexes, allowing us to test whether prenatal stress produces shared or sex-specific outcomes. We then assessed stress-sensitive behavior and endocrine output and quantified stress- and immune-responsive gene expression across key stress-regulatory regions (PFC, hypothalamus, hippocampus, amygdala) immediately after stress (0DPS) and during early recovery (3 days post-stress; 3DPS). We hypothesized that prenatal stress would change how offspring respond to and recover from repeated stress in adulthood, leading to divergent endocrine, behavioral, and transcriptional profiles immediately after stress and during early recovery.

## 2 Materials and methods

### 2.1 Animals and experimental design

C57BL/6J nulliparous female mice (The Jackson Laboratory) were acclimated to the vivarium for 1 week prior to breeding. Animals were maintained on a 12 h light/12 h dark cycle (lights on 06:00–18:00) with ad libitum access to food and water. Females were monogamously paired with C57BL/6J males at 11 weeks of age. Males were singly housed with enrichment prior to pairing. The start of pregnancy (E0.5) was defined by the presence of a vaginal plug detected during daily checks. At E0.5, pregnant dams were separated from breeders and housed individually for the remainder of gestation and through weaning.

All procedures were approved by the Institutional Animal Care and Use Committee (IACUC) at The Ohio State University and conducted in accordance with relevant guidelines.

### 2.2 Experimental design, randomization, and litter handling

Dams were randomly assigned to prenatal stress or control conditions at E10.5. Randomization was performed in R (v4.3.0) using the RandomizeR package, with a new random seed generated each day (random.org). Offspring were weaned at postnatal day 28 (P28) and housed with same-sex littermates (2–4 mice/cage). Prenatal stress-exposed and control offspring were never co-housed.

To limit litter-driven pseudo-replication, no more than one offspring per sex per litter was used for any given endpoint (behavior, 0DPS tissue collection, or 3DPS tissue collection).

### 2.3 Prenatal restraint stress

Beginning at E10.5, pregnant dams in the prenatal stress group underwent restraint stress once daily for 7 consecutive days (E10.5–E16.5). Restraint was performed for 2 h/day and was initiated during the light phase between 9:00 and 11:00 h using a ventilated 50 mL conical tube with holes to permit airflow. Restraint was conducted in the home care environment. Both prenatal stress and control dams were weighed daily during this gestational window. Following the final restraint session on E16.5, dams were left undisturbed until weaning at P28, aside from routine cage changes every 2 weeks with new nesting material provided.

### 2.3 Adult restraint stress

Adult offspring were left undisturbed after weaning until P80–P84. At adulthood, offspring were randomly assigned to an adult restraint stress regimen or adult control condition using the same RandomizeR procedure described above. Adult restraint stress was performed once daily for 7 consecutive days during the light phase (2 h/day) using the same restraint procedure as prenatal stress (ventilated 50 mL conical tube). Adult control offspring were handled by daily weighing during the same period.

### 2.4 Behavioral testing

Behavioral testing was performed under red light during the light phase (09:00–17:00). Mice were acclimated to the testing room for 1 h prior to each test. Behavior was recorded and automatically tracked using EthoVision software (v17, Noldus). All behavioral apparatuses were cleaned between trials with 70% ethanol.

Behavioral testing was conducted in a dedicated cohort that was separate from the 3DPS gene expression cohort to avoid potential behavioral-test–induced stress effects on molecular endpoints. Experimenters were blinded to prenatal and adult condition during behavioral scoring/analysis.

### 2.5 Three-chamber social approach

Social approach was assessed 1 day after the final adult restraint session. The apparatus consisted of a three-chambered box containing two stimulus enclosures as previously described (Gur et al., 2019). Mice were first allowed to freely explore the apparatus for 5 min with both stimulus enclosures empty to habituate and to assess side bias. A novel, age- and sex-matched conspecific of the same strain was then placed into one enclosure and a novel object (beaker cap) was placed into the opposite enclosure; stimulus side assignment was randomized for each trial. Mice were allowed to explore for 10 min.

Social investigation was quantified as time spent in the chamber containing the stimulus mouse. Side bias during habituation was quantified and did not differ across experimental groups.

A social preference index was calculated as:


$$\frac{c\,o\,n\,s\,p\,e\,c\,i\,f\,i\,c\,m\,o\,u\,s\,e\,i\,n\,v\,e\,s\,t\,i\,g\,a\,t\,i\,o\,n\,t\,i\,m\,e-n\,o\,v\,e\,l\,o\,b\,j\,e\,c\,t\,i\,n\,v\,e\,s\,t\,i\,g\,a\,t\,i\,o\,n\,t\,i\,m\,e}{t\,o\,t\,a\,l\,t\,i\,m\,e}$$

### 2.6 Three-chamber social novelty

Immediately following the social approach test, the object was replaced by a second novel, age- and sex-matched conspecific, while the original stimulus mouse remained in place as the “familiar” mouse. Mice were recorded for 10 min to assess preference for social novelty. Social novelty preference was quantified using the same investigation metric described above. A novelty preference index was calculated as:


$$\frac{n\,o\,v\,e\,l\,m\,o\,u\,s\,e\,i\,n\,v\,e\,s\,t\,i\,g\,a\,t\,i\,o\,n\,t\,i\,m\,e-f\,a\,m\,i\,l\,i\,a\,r\,m\,o\,u\,s\,e\,i\,n\,v\,e\,s\,t\,i\,g\,a\,t\,i\,o\,n\,t\,i\,m\,e}{t\,o\,t\,a\,l\,t\,i\,m\,e}$$

### 2.7 Elevated plus maze

Anxiety-like behavior was assessed 2 days after completion of the adult restraint regimen. Mice were placed in the center of the elevated plus maze. The apparatus was elevated 75 cm above the floor and recorded for 10 min. Anxiety-like behavior was quantified as percent time spent in open arms and number of open arm entries.

Behavioral outcomes were analyzed in GraphPad Prism (v10.6.1) using ordinary three-way ANOVA (Type III sums of squares) with Sex (female, male), Prenatal condition (control, prenatal stress), and adult condition (adult control, adult restraint) as fixed between-subject factors. For each behavioral endpoint (e.g., social approach index, social novelty index, percent time in open arms, open arm entries, and distance traveled), significant main effects or interactions were followed by Tukey’s multiple comparisons test comparing all cell means (all Sex × Prenatal × Adult group combinations) with familywise error control (α = 0.05). All tests were two-tailed and adjusted *p*-values are reported.

### 2.8 Tissue collection and processing

Tissue was collected either immediately following the final adult restraint session (0 days post-stress; 0DPS) or 3 days after the final session (3 days post-stress; 3DPS). Mice were euthanized by CO2 asphyxiation. Blood collection was performed immediately after euthanasia via cardiac puncture using an EDTA-coated syringe. Blood was transferred to tubes containing 10 μL EDTA and kept at 4°C until processing. Samples were centrifuged at 2,000 × g for 15 min at 4°C, and plasma was collected and stored at -80°C.

Brains were immediately removed, and dissections were performed on ice. The hippocampus, amygdala, prefrontal cortex, and hypothalamus were collected for gene expression analyses. Tissues were snap frozen and stored at −80°C until RNA isolation.

### 2.9 RNA isolation and cDNA synthesis

Total RNA was isolated using TRIzol reagent (Thermo Fisher Scientific) according to the manufacturer’s protocol. RNA concentration and purity (A260/280) were assessed by NanoDrop spectrophotometry. cDNA was synthesized from 4 ng RNA input using the High-Capacity cDNA Reverse Transcription Kit (Applied Biosystems) according to the manufacturer’s instructions. cDNA was diluted 1:5 prior to quantitative PCR.

### 2.10 Quantitative real-time PCR

Quantitative PCR was performed using TaqMan Fast Advanced Master Mix (Applied Biosystems) on a QuantStudio 5 Real-Time PCR System. Reactions were run in duplicate. TaqMan assay IDs for target genes and reference genes are provided in Supplementary Table 1. Gapdh served as the reference housekeeping gene for ΔΔCt normalization. Relative expression was calculated using the ΔΔCt method with the prenatal control/adult control as the reference. Experimenters were blinded to group during qPCR analysis.

Relative gene expressions were analyzed using the ΔCt method. For each sample, the cycle threshold (Ct) of the target gene was normalized to the Ct of the housekeeping gene *Gapdh*. ΔCt values were calculated as (Ct^Target^ - Ct^Reference^). For graphical representation, data were transformed to fold-change relative to the control group using the 2^(–ΔΔCt)^ method. However, all statistical testing was performed on the ΔCt values.

All analyses were conducted in the R statistical computing environment using the tidyverse, emmeans, and broom packages. Data were analyzed using a three-way linear model (lm) to evaluate the effects of Prenatal Stress, Adult Stress Condition, and Sex, as well as their interactions. Hypothesis testing was conducted using a priori planned contrasts via the emmeans package. We specifically assessed main effect of stress, sex-specific effects, and prenatal interactions. To control the False Discovery Rate (FDR), *p*-values were adjusted using the Benjamini-Hochberg (BH) procedure applied within each gene and statistical family.

### 2.11 Plasma corticosterone ELISA

Plasma corticosterone was quantified using a commercial ELISA kit (Cayman Chemical) according to the manufacturer’s protocol. Plasma samples were diluted 1:15 and run in duplicate. Absorbance was read at 412 nm (Tecan Infinite M Nano). Concentrations were calculated using Cayman’s analysis spreadsheet with a four-parameter logistic (4PL) fit.

Plasma corticosterone concentrations were analyzed in GraphPad Prism using an ordinary three-way ANOVA (Type III sums of squares) with Sex (female, male), Prenatal condition (control, prenatal stress), and Adult condition (adult control, adult restraint) as fixed between-subject factors. Because corticosterone was measured at separate endpoints, 0DPS and 3DPS were analyzed in separate models. When the ANOVA detected significant main effects or interactions, post hoc multiple comparisons were performed using Tukey’s test with familywise error control (α = 0.05), comparing all cell means (i.e., all combinations of Sex × Prenatal condition × Adult condition) within each timepoint. All tests were two-tailed, and adjusted *p*-values from Tukey’s procedure are reported.

## 3 Results

### 3.1 Repeated adult restraint produced a robust, transient corticosterone response

**FIGURE 1:**
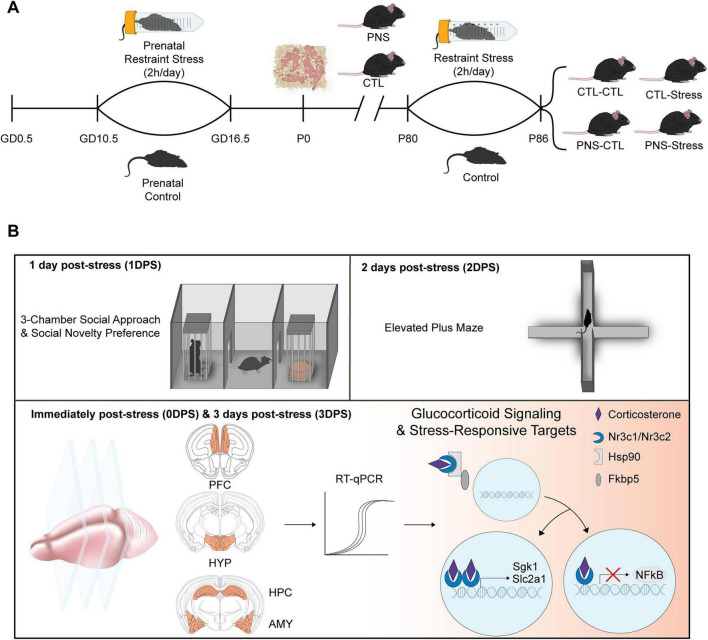
Experimental design overview for prenatal stress, adult stress, and behavioral and molecular assessments. **(A)** Pregnant dams were exposed to prenatal restraint stress (2 h/day) from gestational day (GD) 10.5 to GD16.5 or remained undisturbed (prenatal control). At P80, adult offspring from both prenatal conditions were assigned to either restraint stress (2 h/day) for 1-week or control conditions, generating four experimental groups: CTL–CTL, CTL–Stress, PNS–CTL, and PNS–Stress. **(B)** Social behavior was assessed 1-day post-stress (1DPS) using the three-chamber social approach and social novelty test, and anxiety-like behavior was assessed 2 days post-stress (2DPS) using the elevated plus maze. Tissue collection for gene expression analyses occurred immediately post-stress (0DPS) and 3 days post-stress (3DPS). Prefrontal cortex (PFC), hypothalamus (HYP), hippocampus (HPC), and amygdala (AMY) were dissected for RT-qPCR analysis of glucocorticoid signaling components and stress-responsive targets.

Adult offspring (P80) from prenatal control and prenatal stress litters were assigned to adult control or 1-week restraint stress, yielding four experimental groups, with endocrine and molecular outcomes collected immediately post-stress (0DPS), 3 days post-stress (3DPS), and at equivalent timepoints in non-stressed mice (Figure 1).

**FIGURE 2:**
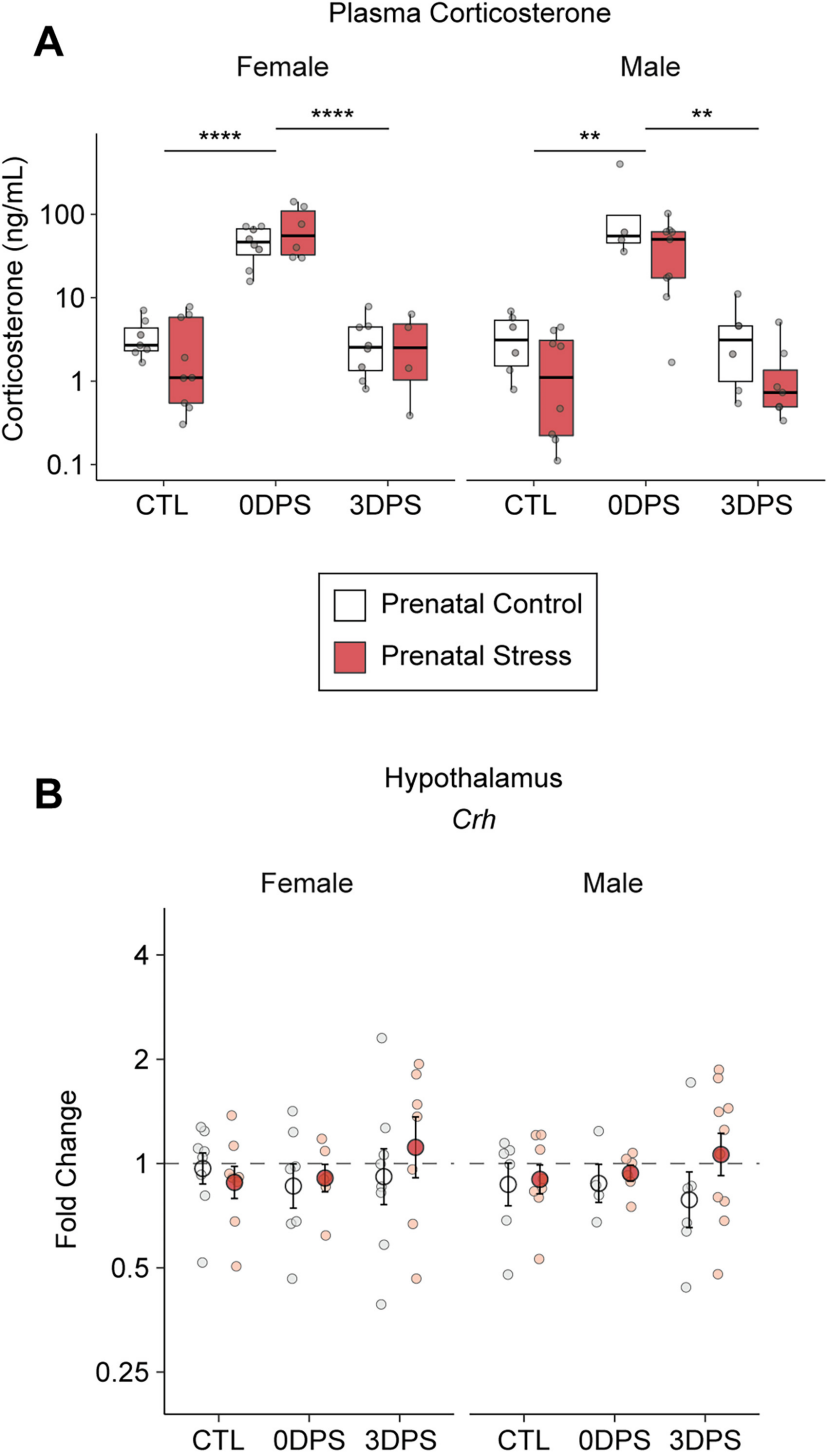
Prenatal stress does not alter the transient corticosterone response to repeated adult restraint. **(A)** Plasma corticosterone concentrations were measured by ELISA in female and male offspring from prenatal control (CTL) and prenatal stress (PNS) groups at baseline (CTL), immediately following adult restraint stress (0 days post-stress; 0DPS), and 3 days post-stress (3DPS). Data are shown on a log scale as box-and-whisker plots with individual data points overlaid. n = 4–9 per group (58 total at 0DPS; 57 total at 3DPS). **(B)** Hypothalamic Crh gene expression was quantified by RT-qPCR at the same time points and is expressed as fold change relative to sex-matched prenatal control animals. Points represent individual animals; larger symbols indicate group means ± SEM. n = 4–10 per group (84 total mice from 33 litters). Statistical significance was assessed within sex using appropriate post hoc comparisons following ANOVA. Asterisks denote significant differences between time points as indicated (**p < 0.01, ****p < 0.0001).

Across sexes and prenatal conditions, repeated adult restraint elicited a significant increase in plasma corticosterone at 0DPS (*p* < 0.0001) that decreased to comparable levels with control animals by 3DPS (*p* < 0.0001) (Figure 2A). In contrast, hypothalamic *Crh* gene expression was relatively stable across time points, showing no significant differences following repeated adult stress or from prenatal stress in either sex (Figure 2B).

### 3.2 Prenatal stress selectively modified social novelty behavior after stress in adulthood, without affecting anxiety-like behavior

**FIGURE 3:**
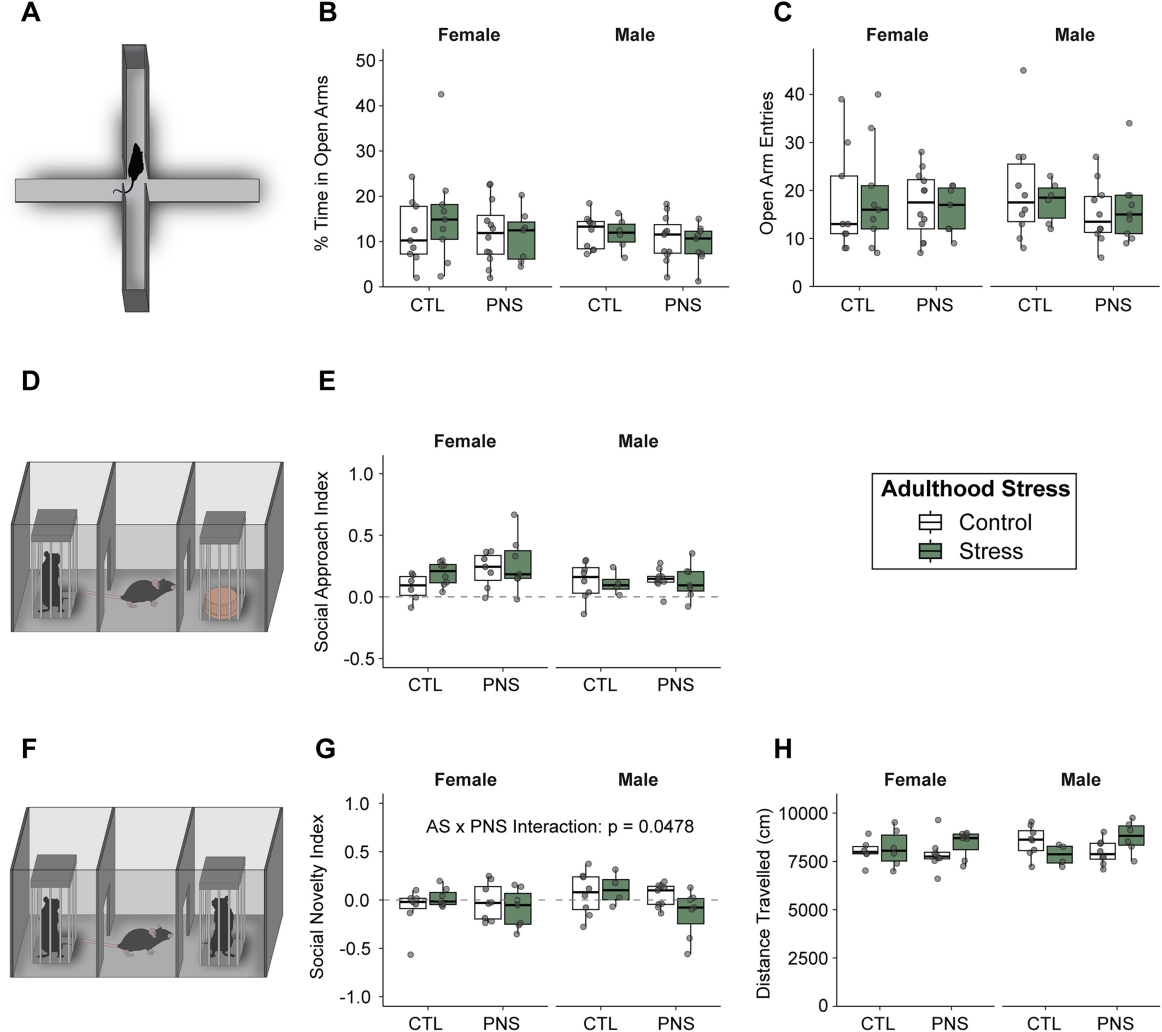
Prenatal stress modulates adult stress effects on social novelty, without altering anxiety-like behavior. **(A)** Schematic of the elevated plus maze (EPM) used to assess anxiety-like behavior. **(B,C)** Percent time spent in the open arms **(B)** and number of open arm entries **(C)** during the EPM, plotted separately for female and male mice. **(D)** Schematic of the three-chamber social interaction apparatus. **(E)** Social approach index and **(G)** social novelty index measured during the three-chamber test, plotted by prenatal condition (CTL, prenatal control; PNS, prenatal stress), adult condition (control or stress), and sex. **(F)** Schematic illustrating the social novelty phase of the three-chamber task. **(H)** Total distance traveled during behavioral testing, shown as a measure of general locomotor activity. *n* = 4–7 per group (58 total from 30 litters) for social behavior testing. *n* = 6–12 per group (73 total from 33 litters) for EPM testing. Data are shown as box-and-whisker plots with individual data points overlaid; boxes represent the interquartile range with median indicated. Adulthood stress is denoted by color (white, control; green, stress). Dashed horizontal lines indicate zero for index measures. A significant adulthood stress × prenatal stress interaction was detected for social novelty preference by three-way ANOVA (sex × prenatal stress × adult stress; *p* = 0.0478).

To determine whether prenatal stress altered behavioral sensitivity to adult stress, we quantified anxiety-like behavior in the elevated plus maze (EPM) and social behavior in the three-chamber assay 1–2 days after completion of the adult stress regimen (Figures 1B, 3). In the EPM, there were no significant differences in the percent time nor the entries in the open arms due to adulthood stress, prenatal stress, sex, or their interactions (Figures 3B,C). In the three-chamber task, social approach index was not affected (Figure 3E), whereas social novelty preference demonstrated a significant interaction between prenatal stress and adulthood stress. Specifically, three-way ANOVA detected a significant prenatal stress × adult stress interaction for social novelty index (*p* = 0.0478), indicating that prenatal stress altered how adult stress impacted novelty preference (Figure 3G). There were no differences in locomotor activity (distance traveled) suggesting no confounding effects by activity differences (Figure 3H).

### 3.3 Adult stress induced a broad, region-spanning glucocorticoid-signaling transcriptional signature at 0DPS that largely resolved by 3DPS

**FIGURE 4:**
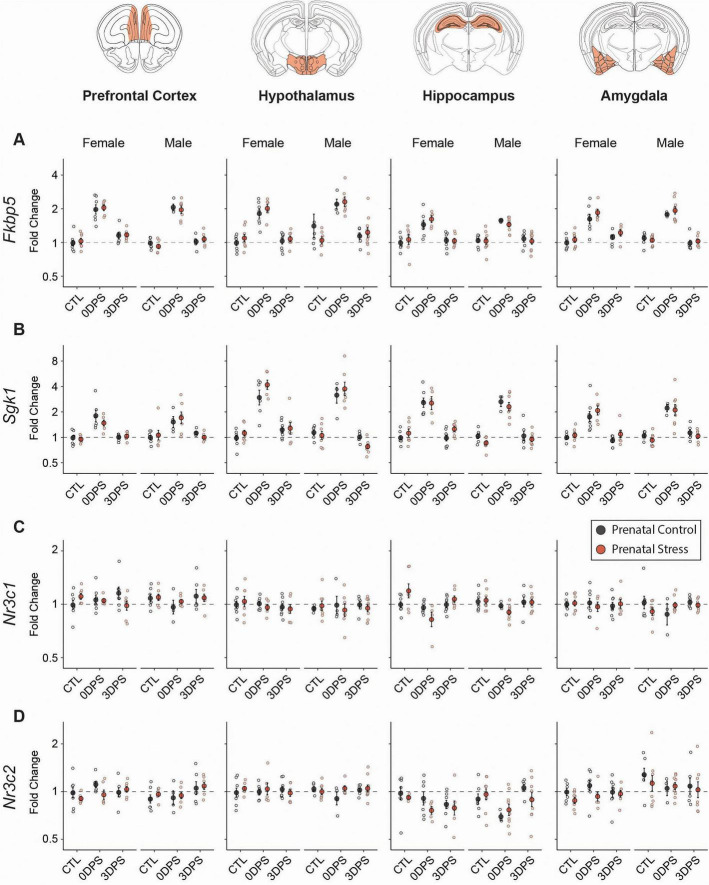
Adult stress evokes a broad, transient glucocorticoid-responsive transcriptional program across stress-regulatory brain regions. RT-qPCR analysis of **(A)** *Fkbp5*, **(B)** *Sgk1*, **(C)** *Nr3c1*, and **(D)** *Nr3c2* expression in the prefrontal cortex, hypothalamus, hippocampus, and amygdala of female and male mice. Animals were from prenatal control (black) or prenatal stress (red) groups and were assessed at baseline (CTL), immediately following 7 consecutive days of adult restraint stress (0 days post-stress; 0DPS), or 3 days post-stress (3DPS). Data are expressed as fold change relative to sex-matched prenatal control animals at baseline and plotted on a log2 scale. Points represent individual animals; larger symbols indicate mean ± SEM. Dashed lines indicate baseline expression (fold change = 1). Sample size varied by region: amygdala 3–9 (total 86 mice from 32 litters), hippocampus 3–9 (85 mice from 33 litters), hypothalamus 4–10 (84 mice from 33 litters), PFC 4–8 (79 mice from 30 litters).

We next tested whether prenatal stress altered the magnitude or persistence of adult stress–evoked transcriptional responses across stress-regulatory circuitry (PFC, hypothalamus, hippocampus, amygdala) (Figure 4). Consistent with robust HPA-axis activation, adult stress produced strong induction of canonical glucocorticoid-responsive targets *Fkbp5* and *Sgk1* 0DPS across all regions in both sexes (*p* < 0.001 for all regions and sex; Table 1). By 3DPS, these glucocorticoid-signaling transcriptional responses were no longer significant as main effects of adult stress across regions except for a persistent significant small increase in *Fkbp5* in female PFC and amygdala (Figures 4A,B and Table 2), indicating recovery of this transcriptional program largely by 3DPS.

**TABLE 1:** Sex-stratified adult stress effects and prenatal × adult stress interactions on qPCR targets at 0 days post-stress (0DPS) across brain regions.

Brain region/Gene	Stress Effect (0DPS)		Interaction (0DPS)	
	Female	Male	Female	Male
Amygdala				
Bdnf	*p* = 0.942 (0.998)	*p* = 0.172 (0.668)	*p* = 0.822 (0.941)	*p* = 0.173 (0.778)
Fkbp5	*p* < 0.001 (< 0.001)*	*p* < 0.001 (<0.001)*	*p* = 0.570 (0.733)	*p* = 0.361 (0.541)
Il1b	*p* = 0.089 (0.179)	*p* < 0.001 (0.004)*	*p* = 0.340 (0.611)	*p* = 0.493 (0.715)
Il6	*p* = 0.003 (0.009)*	*p* = 0.016 (0.031)*	*p* = 0.638 (0.879)	*p* = 0.512 (0.838)
Nr3c1	*p* = 0.790 (0.947)	*p* = 0.551 (0.947)	*p* = 0.505 (0.913)	*p* = 0.044 (0.652)
Nr3c2	*p* = 0.364 (0.728)	*p* = 0.204 (0.613)	*p* = 0.831 (0.887)	*p* = 0.406 (0.887)
Sgk1	*p* < 0.001 (<0.001)*	*p* < 0.001 (<0.001)*	*p* = 0.571 (0.796)	*p* = 0.735 (0.914)
Slc2a1	*p* < 0.001 (<0.001)*	*p* < 0.001 (<0.001)*	*p* = 0.562 (0.633)	*p* = 0.103 (0.205)
Tnf	*p* < 0.001 (0.002)*	*p* = 0.070 (0.104)	*p* = 0.440 (0.726)	*p* = 0.571 (0.791)
Hippocampus				
Bdnf	*p* = 0.008 (0.035)*	*p* = 0.012 (0.035)*	*p* = 0.908 (0.908)	*p* = 0.212 (0.484)
Fkbp5	*p* < 0.001 (<0.001)*	*p* < 0.001 (<0.001)*	*p* = 0.760 (0.864)	*p* = 0.620 (0.864)
Il1b	*p* < 0.001 (0.002)*	*p* = 0.100 (0.149)	*p* = 0.301 (0.492)	*p* = 0.203 (0.460)
Il6	*p* = 0.412 (0.797)	*p* = 0.732 (0.824)	*p* = 0.697 (0.810)	*p* = 0.460 (0.764)
Nr3c1	*p* < 0.001 (0.001)*	*p* = 0.072 (0.143)	*p* = 0.002 (0.014)*	*p* = 0.373 (0.746)
Nr3c2	*p* = 0.148 (0.221)	*p* = 0.012 (0.035)*	*p* = 0.522 (0.636)	*p* = 0.867 (0.919)
Sgk1	*p* < 0.001 (<0.001)*	*p* < 0.001 (<0.001)*	*p* = 0.483 (0.711)	*p* = 0.806 (0.943)
Slc2a1	*p* < 0.001 (<0.001)*	*p* < 0.001 (<0.001)*	*p* = 0.327 (0.438)	*p* = 0.341 (0.438)
Tnf	*p* < 0.001 (<0.001)*	*p* = 0.002 (0.004)*	*p* = 0.506 (0.651)	*p* = 0.484 (0.651)
Hypothalamus				
Bdnf	*p* = 0.349 (0.698)	*p* = 0.912 (0.912)	*p* = 0.119 (0.715)	*p* = 0.597 (0.851)
Crh	*p* = 0.758 (0.962)	*p* = 0.880 (0.962)	*p* = 0.607 (0.967)	*p* = 0.913 (0.967)
Fkbp5	*p* < 0.001 (<0.001)*	*p* < 0.001 (<0.001)*	*p* = 0.970 (0.970)	*p* = 0.128 (0.231)
Il1b	*p* = 0.039 (0.077)	*p* < 0.001 (<0.001)*	*p* = 0.334 (0.536)	*p* = 0.384 (0.536)
Il6	*p* = 0.520 (0.520)	*p* = 0.124 (0.372)	*p* = 0.765 (0.848)	*p* = 0.465 (0.825)
Nr3c1	*p* = 0.556 (0.926)	*p* = 0.926 (0.926)	*p* = 0.402 (0.918)	*p* = 0.419 (0.918)
Nr3c2	*p* = 0.948 (0.948)	*p* = 0.432 (0.948)	*p* = 0.857 (0.966)	*p* = 0.115 (0.828)
Sgk1	*p* < 0.001 (<0.001)*	*p* < 0.001 (<0.001)*	*p* = 0.365 (0.469)	*p* = 0.330 (0.457)
Slc2a1	*p* < 0.001 (0.001)*	*p* = 0.015 (0.023)*	*p* = 0.643 (0.827)	*p* = 0.124 (0.265)
Tnf	*p* = 0.015 (0.034)*	*p* = 0.001 (0.007)*	*p* = 0.484 (0.654)	*p* = 0.390 (0.585)
Prefrontal cortex				
Bdnf	*p* = 0.357 (0.357)	*p* = 0.306 (0.357)	*p* = 0.929 (0.962)	*p* = 0.349 (0.572)
Fkbp5	*p* < 0.001 (<0.001)*	*p* < 0.001 (<0.001)*	*p* = 0.973 (0.973)	*p* = 0.923 (0.973)
Il1b	*p* = 0.005 (0.007)*	*p* < 0.001 (0.001)*	*p* = 0.994 (0.994)	*p* = 0.066 (0.119)
Il6	*p* = 0.017 (0.051)	*p* = 0.908 (0.908)	*p* = 0.954 (0.954)	*p* = 0.736 (0.954)
Nr3c1	*p* = 0.913 (0.913)	*p* = 0.155 (0.465)	*p* = 0.274 (0.616)	*p* = 0.603 (0.723)
Nr3c2	*p* = 0.135 (0.270)	*p* = 0.997 (0.997)	*p* = 0.579 (0.868)	*p* = 0.782 (0.965)
Sgk1	*p* < 0.001 (<0.001)*	*p* < 0.001 (<0.001)*	*p* = 0.380 (0.527)	*p* = 0.792 (0.838)
Slc2a1	*p* < 0.001 (<0.001)*	*p* < 0.001 (<0.001)*	*p* = 0.077 (0.154)	*p* = 0.369 (0.537)
Tnf	*p* < 0.001 (< 0.001)*	*p* = 0.033 (0.049)*	*p* = 0.510 (0.765)	*p* = 0.823 (0.837)

Values are shown as p (FDR-adjusted p). Significant results (adjusted *p* < 0.05) are shown in bold and marked with an asterisk.

**TABLE 2:** Sex-stratified adult stress effects and prenatal × adult stress interactions on qPCR targets at 3 days post-stress (3DPS) across brain regions.

Brain region/gene	Stress effect (3DPS)		Interaction (3DPS)	
	Female	Male	Female	Male
Amygdala				
Bdnf	*p* = 0.943 (0.998)	*p* = 0.223 (0.668)	*p* = 0.631 (0.941)	*p* = 0.011 (0.115)
Fkbp5	*p* = 0.039 (0.047)*	*p* = 0.300 (0.300)	*p* = 0.777 (0.797)	*p* = 0.483 (0.669)
Il1b	*p* = 0.447 (0.536)	*p* = 0.747 (0.747)	*p* = 0.146 (0.376)	*p* = 0.618 (0.715)
Il6	*p* = 0.959 (0.959)	*p* = 0.370 (0.444)	*p* = 0.965 (0.996)	*p* = 0.226 (0.452)
Nr3c1	*p* = 0.768 (0.947)	*p* = 0.375 (0.947)	*p* = 0.924 (0.986)	*p* = 0.443 (0.913)
Nr3c2	*p* = 0.558 (0.838)	*p* = 0.123 (0.613)	*p* = 0.555 (0.887)	*p* = 0.662 (0.887)
Sgk1	*p* = 0.741 (0.741)	*p* = 0.311 (0.373)	*p* = 0.536 (0.796)	*p* = 0.864 (0.914)
Slc2a1	*p* = 0.849 (0.849)	*p* = 0.709 (0.849)	*p* = 0.350 (0.479)	*p* = 0.124 (0.223)
Tnf	*p* = 0.172 (0.206)	*p* = 0.892 (0.892)	*p* = 0.922 (0.964)	*p* = 0.845 (0.951)
Hippocampus				
Bdnf	*p* = 0.124 (0.186)	*p* = 0.780 (0.780)	*p* = 0.805 (0.852)	*p* = 0.377 (0.566)
Fkbp5	*p* = 0.874 (0.874)	*p* = 0.799 (0.874)	*p* = 0.565 (0.864)	*p* = 0.766 (0.864)
Il1b	*p* = 0.013 (0.038)*	*p* = 0.559 (0.559)	*p* = 0.383 (0.496)	*p* = 0.331 (0.496)
Il6	*p* = 0.824 (0.824)	*p* = 0.531 (0.797)	*p* = 0.501 (0.764)	*p* = 0.056 (0.250)
Nr3c1	*p* = 0.282 (0.338)	*p* = 0.771 (0.771)	*p* = 0.283 (0.638)	*p* = 0.849 (0.956)
Nr3c2	*p* = 0.065 (0.130)	*p* = 0.585 (0.702)	*p* = 0.956 (0.956)	*p* = 0.140 (0.370)
Sgk1	*p* = 0.568 (0.568)	*p* = 0.565 (0.568)	*p* = 0.513 (0.711)	*p* = 0.600 (0.772)
Slc2a1	*p* = 0.894 (0.894)	*p* = 0.063 (0.076)	*p* = 0.466 (0.559)	*p* = 0.577 (0.611)
Tnf	*p* = 0.196 (0.235)	*p* = 0.726 (0.726)	*p* = 0.842 (0.891)	*p* = 0.730 (0.822)
Hypothalamus				
Bdnf	*p* = 0.233 (0.698)	*p* = 0.660 (0.791)	*p* = 0.569 (0.851)	*p* = 0.842 (0.898)
Crh	*p* = 0.511 (0.962)	*p* = 0.824 (0.962)	*p* = 0.293 (0.967)	*p* = 0.346 (0.967)
Fkbp5	*p* = 0.903 (0.903)	*p* = 0.823 (0.903)	*p* = 0.775 (0.930)	*p* = 0.069 (0.139)
Il1b	*p* = 0.091 (0.110)	*p* = 0.013 (0.040)*	*p* = 0.878 (0.878)	*p* = 0.128 (0.383)
Il6	*p* = 0.199 (0.398)	*p* = 0.313 (0.469)	*p* = 0.365 (0.821)	*p* = 0.848 (0.848)
Nr3c1	*p* = 0.223 (0.926)	*p* = 0.874 (0.926)	*p* = 0.520 (0.918)	*p* = 0.475 (0.918)
Nr3c2	*p* = 0.861 (0.948)	*p* = 0.764 (0.948)	*p* = 0.242 (0.828)	*p* = 0.552 (0.828)
Sgk1	*p* = 0.113 (0.113)	*p* = 0.072 (0.086)	*p* = 0.683 (0.683)	*p* = 0.443 (0.498)
Slc2a1	*p* = 0.910 (0.910)	*p* = 0.238 (0.285)	*p* = 0.890 (0.986)	*p* = 0.250 (0.450)
Tnf	*p* = 0.350 (0.350)	*p* = 0.017 (0.034)*	*p* = 0.508 (0.654)	*p* = 0.807 (0.949)
Prefrontal cortex				
Bdnf	*p* = 0.234 (0.350)	*p* = 0.010 (0.031)*	*p* = 0.024 (0.108)	*p* = 0.750 (0.900)
Fkbp5	*p* = 0.017 (0.020)*	*p* = 0.139 (0.139)	*p* = 0.818 (0.956)	*p* = 0.332 (0.497)
Il1b	*p* = 0.263 (0.316)	*p* = 0.698 (0.698)	*p* = 0.027 (0.092)	*p* = 0.930 (0.985)
Il6	*p* = 0.846 (0.908)	*p* = 0.583 (0.908)	*p* = 0.189 (0.815)	*p* = 0.813 (0.954)
Nr3c1	*p* = 0.756 (0.913)	*p* = 0.857 (0.913)	*p* = 0.012 (0.223)	*p* = 0.764 (0.809)
Nr3c2	*p* = 0.223 (0.334)	*p* = 0.020 (0.094)	*p* = 0.275 (0.549)	*p* = 0.759 (0.965)
Sgk1	*p* = 0.575 (0.690)	*p* = 0.741 (0.741)	*p* = 0.700 (0.787)	*p* = 0.334 (0.527)
Slc2a1	*p* = 0.656 (0.787)	*p* = 0.795 (0.795)	*p* = 0.426 (0.548)	*p* = 0.538 (0.605)
Tnf	*p* = 0.122 (0.147)	*p* = 0.274 (0.274)	*p* = 0.246 (0.425)	*p* = 0.639 (0.795)

Values are shown as p (FDR-adjusted p). Significant results (adjusted *p* < 0.05) are shown in bold and marked with an asterisk.

In contrast to these glucocorticoid-signaling targets, GR/MR-encoding transcripts were comparatively stable. Adult stress effects were restricted to the hippocampus and were dependent on sex. In females, hippocampal Nr3c1 at 0DPS showed a significant prenatal × adult stress interaction (FDR = 0.0141; Table 2). Post-hoc testing indicated that adult stress decreased *Nr3c1* specifically in PNS offspring (FDR = 0.0002; Supplementary Data 1) whereas no stress effect was detected in prenatal controls (FDR = 0.797). In males, adult stress was associated with lower hippocampal *Nr3c2* at 0DPS (FDR = 0.035), followed by an increase from 0DPS to 3DPS (FDR = 0.021). No significant adult stress effects were detected for *Nr3c1* or *Nr3c2* in the amygdala, hypothalamus, or prefrontal cortex at 0DPS or 3DPS (all FDR > 0.05; Table 2).

### 3.4 Repeated restraint stress decreased pro-inflammatory cytokine transcripts immediately after stress, with limited persistence and selective prenatal modulation during recovery

**FIGURE 5:**
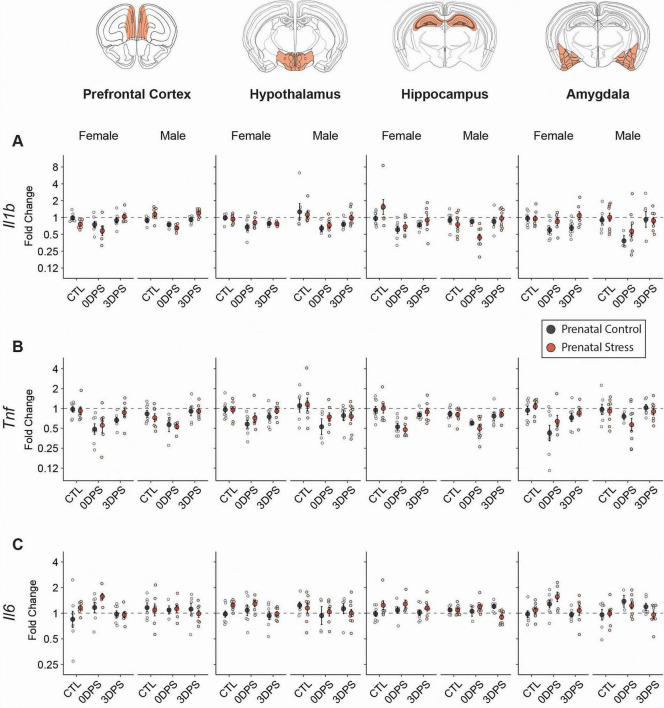
Repeated restraint stress suppresses Il1b and Tnf immediately following stress with limited persistence 3DPS. RT-qPCR analysis of **(A)** *Il1b*, **(B)** *Tnf*, and **(C)** *Il6* mRNA expression in the prefrontal cortex, hypothalamus, hippocampus, and amygdala of female and male mice from prenatal control (black) and prenatal stress (red) groups. Animals were assessed at baseline (CTL), immediately following adult restraint stress (0 days post-stress; 0DPS), or 3 days post-stress (3DPS). Data are expressed as fold change relative to sex-matched prenatal control animals at baseline and plotted on a log2-scaled y-axis. Individual points represent biological replicates; larger symbols indicate mean ± SEM. Dashed horizontal lines denote baseline expression (fold change = 1). Sample size varied by region: amygdala 3–9 (total 86 mice from 32 litters), hippocampus 3–9 (85 mice from 33 litters), hypothalamus 4–10 (84 mice from 33 litters), PFC 4–8 (79 mice from 30 litters).

We evaluated changes in the neuroimmune response by determining changes in *Il1b*, *Tnf*, and *Il6* expression across regions (Figures 5A–C). *Il1b* and *Tnf* showed overlapping patterns consistent with an acute cytokine blunting at 0DPS, with *Tnf* exhibiting the broader response. In females, adult stress decreased *Il1b* in hippocampus (FDR = 0.00199) and PFC (FDR = 0.00696), while *Tnf* decreased across all regions (amygdala FDR = 0.00211; hippocampus FDR < 0.001; hypothalamus FDR = 0.0341; PFC FDR < 0.001; Table 1). In males, adult stress decreased *Il1b* in hypothalamus (FDR < 0.001), PFC (FDR = 0.00106), and amygdala (FDR = 0.004) and decreased *Tnf* in hippocampus (FDR = 0.00384), hypothalamus (FDR = 0.00715), and PFC (FDR = 0.0495). By 3DPS, these cytokine inductions largely returned toward control levels, with persistence limited to female hippocampal *Il1b* (FDR = 0.0381) and male hypothalamic *Il1b* and *Tnf* (FDR = 0.0397 and 0.0341).*Il6* showed a more restricted pattern and in the opposite direction. Stress in adulthood increased amygdala *Il6* at 0DPS in both sexes (female FDR = 0.00913; male FDR = 0.0313), with no other stress effects after correction. No sex-stratified prenatal × adult stress interactions were detected for these cytokines after FDR correction (Table 2).

### 3.5 Downstream targets of stress signaling show transient and region-specific regulation after adult stress

**FIGURE 6:**
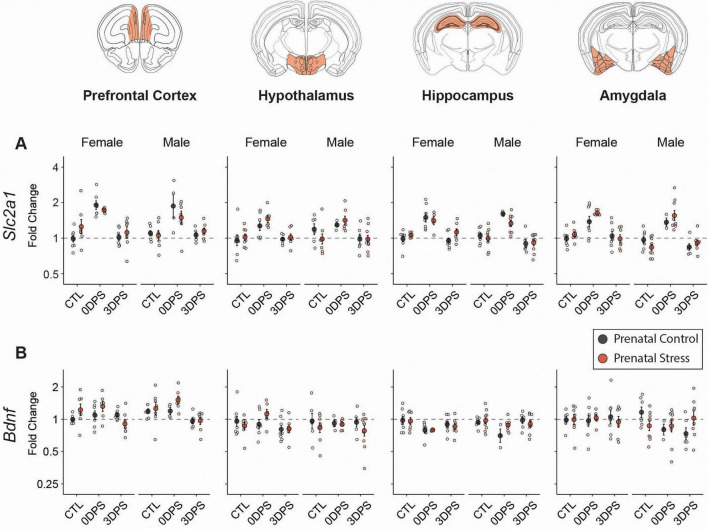
Downstream targets of stress signaling show transient, region-specific regulation after adult stress. RT-qPCR analysis of **(A)** *Slc2a1* **(B)** and *Bdnf* gene expression in the prefrontal cortex, hypothalamus, hippocampus, and amygdala of female and male mice from prenatal control (black) and prenatal stress (red) groups. Animals were assessed at baseline (CTL), immediately following adult restraint stress (0 days post-stress; 0DPS), or 3 days post-stress (3DPS). Data are expressed as fold change relative to sex-matched prenatal control animals at baseline and plotted on a log2-scaled y-axis. Individual points represent biological replicates; larger symbols indicate mean ± SEM. Dashed horizontal lines denote baseline expression (fold change = 1). Sample size varied by region: amygdala 3–9 (total 86 mice from 32 litters), hippocampus 3–9 (85 mice from 33 litters), hypothalamus 4–10 (84 mice from 33 litters), PFC 4–8 (79 mice from 30 litters).

*Slc2a1* (Figure 6A) was increased at 0DPS in stressed animals across all regions in both sexes (all FDR < 0.05) Prenatal stress did not significantly moderate the acute *Slc2a1* stress response (all prenatal × adult stress interaction FDR > 0.15). By 3DPS, *Slc2a1* levels were not detectably different between stressed and control animals in any region or sex (all FDR > 0.05), consistent with a return toward control levels.

Bdnf (Figure 6B) showed selective effects rather than a global response. In the hippocampus, adult stress was associated with lower *Bdnf* at 0DPS in both females and males (both FDR = 0.0349), with no remaining stress effect at 3DPS (female FDR = 0.186; male FDR = 0.780). In contrast, in the PFC, adult stress increased *Bdnf* only in males at 3DPS (FDR = 0.0309), with no significant stress effect in females at either timepoint. Amygdala and hypothalamus *Bdnf* did not show significant adult-stress effects after correction (all FDR > 0.05), and no prenatal × adult stress interactions survived FDR correction for *Bdnf* (Table 2). In female PFC, the prenatal × adult stress interaction for Bdnf at 3DPS was nominal (*p* = 0.024) but did not survive FDR correction (FDR ≈ 0.108). Exploratory *post-hoc* analysis suggested a time-dependent decrease in stressed PNS females (3DPS vs. 0DPS, FDR ≈ 0.0376), with no corresponding change in prenatal controls (Supplementary Data 1).

## 4 Discussion

We used a 2 × 2 prenatal stress × adult stress design in both sexes to test whether prenatal stress shifts the magnitude or recovery of stress-evoked endocrine, behavioral, and transcriptional responses across stress-regulatory brain regions. Repeated adult restraint stress elicited a robust but transient corticosterone increase with a robust induction of canonical glucocorticoid-responsive genes (*Sgk1, Fkbp5, Slc2a1*) and inhibition of pro-inflammatory cytokines (*Il1b and Tnf*) immediately following stress that largely resolved by 3 days post-stress. Prenatal stress did not produce anxiety-like behavioral changes but altered stress-related outcomes in a selective, context-dependent manner, including a prenatal × adult stress interaction on social novelty preference and region/sex/timepoint-specific modulation of gene expression. The absence of anxiety-like behaviors following prenatal stress suggests that its impact on adult stress responses is nuanced and context-dependent, manifesting primarily in altered social novelty preference and specific patterns of gene regulation rather than broad behavioral deficits. These findings indicate that prenatal stress may predispose individuals to subtle, region- and sex-specific changes in stress-related adaptability rather than overt behavioral disruptions.

Despite the robust physiological response to adult restraint, prenatal stress did not alter the scale of the corticosterone surge or hypothalamic *Crh* expression in either sex. This absence of HPA-axis potentiation contrasts with prior rodent work demonstrating heightened corticosterone responses in PNS offspring following acute, short-duration stressors, such as brief restraint (Mueller and Bale, 2008; Brunton and Russell, 2010) and forced swim stress (Enayati et al., 2020). Similarly, while hypothalamic *Crh* expression remained stable in our model, other studies have reported elevated *Crh* in female prenatal stress offspring (García-Cáceres et al., 2010; Zohar and Weinstock, 2011) or within the central amygdala of males (Mueller and Bale, 2008). However, our observation of stable hypothalamic *Crh* aligns with other reports showing no differences caused by prenatal stress in the hypothalamus (Brunton and Russell, 2010). These discrepancies are likely attributable to the severity and duration of the adult stressor. While acute stressors may reveal subtle PNS-driven sensitizations, our 7-day repeated regimen may have driven corticosterone levels to a physiological “ceiling” in both groups, potentially obscuring latent vulnerabilities. This interpretation is supported by our previous work, where male PNS offspring exhibited a potentiated corticosterone response and elevated cortical *Crh* following a milder social interaction stressor (Gur et al., 2019). Together, these data suggest that PNS-induced HPA-axis phenotypes are highly stressor- and context-dependent, emerging primarily when the HPA-axis is not driven close to its physiological limit.

SGK1 is a glucocorticoid responsive kinase that has been repeatedly implicated in stress related psychopathology. In humans, SGK1 mRNA is elevated in peripheral blood of drug free depressed patients (Anacker et al., 2013), with higher hippocampal SGK1 expression also found in depressed suicide decedents with the highest expression in those who experienced early life adversity (Millette et al., 2025). Rodent models complement these clinical findings, with increased hippocampal Sgk1 reported after chronic stress, and in adult offspring exposed to prenatal stress (Anacker et al., 2013) and early life stress (Millette et al., 2025). Here, 1 week of repeated restraint stress robustly increased Sgk1 expression immediately after stress across brain regions in both sexes, and this induction was no longer evident by 3DPS with no modulation caused by prenatal stress exposure. Given that Sgk1 transcription is directly regulated by glucocorticoid receptor activation, the lack of prenatal stress-dependent differences in Sgk1 expression parallels our findings of equivalent corticosterone responses in these animals. Additionally, while some prior reports indicate elevated basal hippocampal Sgk1 in prenatally stressed adults (Anacker et al., 2013), our observation of comparable expression between prenatal stress and control suggests that transcriptional dysregulation of this target may depend on species-specific factors or the type and timing of the prenatal insult.

FKBP5 is a cochaperone of the glucocorticoid receptor (GR) that regulates HPA-axis negative feedback and has been widely implicated in the etiology of mood disorders. In humans, FKBP5 expression is elevated in the hippocampus and prefrontal cortex of suicide decedents, where it correlates with decreased methylation (Rizavi et al., 2023), while another study had found decreased expression in the amygdala (Pérez-Ortiz et al., 2013). Consistent with a role in maladaptive stress responses, chronic mild stress in rodents increases *Fkbp5* expression, a phenotype reversed by antidepressant treatment (Guidotti et al., 2013). While prenatal stress has been shown to increase basal *Fkbp5* expression in the PFC of vulnerable adolescent male mice without affecting the acute stress response (Orso et al., 2025), it remained unclear whether these perturbations persist into adulthood or manifest during repeated stress challenge. In the present study, repeated restraint stress produced a robust, ubiquitous induction of *Fkbp5* across brain regions in both sexes, confirming the sensitivity of this target to our stress paradigm. However, we detected no impact of prenatal stress on basal or stress-evoked *Fkbp5* levels in adulthood. These findings suggest that the PNS-induced upregulation of *Fkbp5* observed in adolescence may be a transient developmental phenotype that normalizes by adulthood, or that the strong induction driven by repeated restraint overrides subtle programming effects.

In humans, prenatal psychosocial stress is associated with increased NR3C1 promoter methylation (Palma-Gudiel et al., 2015), and postmortem studies report reduced GR expression in the prefrontal cortex (Rizavi et al., 2023) and amygdala (Pérez-Ortiz et al., 2013) but not hippocampus in suicide cohorts, consistent with region-specific GR dysregulation. Rodent prenatal stress models show similarly heterogeneous outcomes, including reduced hippocampal GR with increased promoter methylation in early-gestation stressed male offspring (Mueller and Bale, 2008) and reduced GR in the mPFC and hippocampus in mid-late gestation stressed offspring that was not further altered by chronic stress later in life (Green et al., 2011). However, these findings are not consistent across prenatal stress models as others have shown no change in hippocampal GR but decreased MR expression (Brunton and Russell, 2010).

Our data extends prior work by testing whether prenatal stress modifies GR and MR transcript regulation during a 1-week repeated restraint challenge across acute and early recovery phases. Overall, GR/MR-encoding transcripts were comparatively stable across stress-regulatory regions, with detectable stress effects largely confined to the hippocampus and differing by sex. In females, hippocampal *Nr3c1* at 0DPS showed a significant prenatal × adult stress interaction, driven by an adult stress–induced decrease in *Nr3c1* specifically in prenatally stressed offspring, with no corresponding stress effect in prenatal controls. In males, adult stress was associated with reduced hippocampal *Nr3c2* at 0DPS followed by a rebound by 3DPS, consistent with time-dependent MR transcript dynamics during early recovery. Notably, we did not observe significant adult-stress effects on *Nr3c1* or *Nr3c2* in the amygdala, hypothalamus, or prefrontal cortex at either timepoint after correction. Together, these findings argue against a global prenatal shift in basal GR/MR transcript abundance and instead indicate that prenatal stress can selectively bias hippocampal receptor transcript regulation during acute stress exposure and early recovery. Functionally, reduced hippocampal *Nr3c1* during acute stress may indicate diminished local glucocorticoid sensitivity, which could in turn alter the timing or magnitude of downstream stress-responsive transcription within this region without necessarily producing detectable changes in circulating corticosterone.

We had hypothesized that prenatal stress would exaggerate the adult neuroimmune response based on prior evidence that stress can prime immune and microglial responses to later perturbations (Frank et al., 2007; Frank et al., 2012; Weber et al., 2019; Ferle et al., 2020; Barrett et al., 2021) and that prenatal stress induces persistent alterations in microglial phenotype and inflammatory reactivity (Diz-Chaves et al., 2012; Diz-Chaves et al., 2013). Contrary to that expectation, prenatal stress did not produce a generalized enhancement of stress-evoked cytokine expression under this repeated restraint paradigm. One possible explanation is that the similar, robust corticosterone response across prenatal conditions may have produced a ceiling effect that masked subtler priming-dependent differences. Rather, repeated restraint broadly suppressed *Il1b* and *Tnf* across regions, consistent with acute glucocorticoid-mediated attenuation of canonical inflammatory signaling (Auphan et al., 1995), while *Il6* remained regionally inducible in the amygdala. Together, these findings suggest that stress-induced regulation of *Il6* in the brain is at least partly dissociable from that of *Il1b* and *Tnf*, with greater regional specificity. Given that IL-6 signaling within stress-regulatory regions has been implicated in stress-induced social avoidance and anxiety-like and depressive-like behavior in rodent models (Chourbaji et al., 2006; Niraula et al., 2019), the selective amygdala induction observed here may have functional significance that warrants further investigation.

Brain-derived neurotrophic factor (BDNF) is a critical mediator of neural plasticity that is highly susceptible to transcriptional suppression by stress (Notaras and van den Buuse, 2020). In prenatal stress, BDNF has been shown to have increased methylation with a concomitant decrease in Bdnf expression in the hippocampus, amygdala, and PFC (Boersma et al., 2014; Dong et al., 2015). In humans, this disrupted BDNF methylation is evident in the offspring of mothers experiencing depression (Braithwaite et al., 2015). Consistent with stress-induced modulation of neurotrophic signaling, repeated adult restraint was associated with an acute reduction of *Bdnf* in the hippocampus at 0DPS in both sexes, with no detectable stress effect by 3DPS, indicating a transient hippocampal response. Outside the hippocampus, *Bdnf* regulation was region- and sex-dependent rather than global. In the PFC, adult stress increased *Bdnf* selectively in males at 3DPS, whereas females showed no significant adult-stress effect at either timepoint. In the amygdala and hypothalamus, we did not detect adult-stress effects on *Bdnf*, and no prenatal × adult stress interactions survived FDR correction in any region. Notably, the nominal prenatal × adult stress interaction in female PFC at 3DPS did not meet FDR significance, but exploratory time-course contrasts suggested a stress-induced decline from 0DPS to 3DPS in prenatally stressed females that was not observed in prenatal controls. Taken together, these data support a robust, transient hippocampal *Bdnf* suppression after repeated stress and suggest that any prenatal stress–related modulation of *Bdnf* timing is subtle and may be most apparent in female PFC during early recovery. Given the central role of hippocampal BDNF in activity-dependent synaptic plasticity, even transient suppression of *Bdnf* expression during the post-stress period may reflect a temporary reduction in neurotrophic support for adaptive remodeling in this region, with potential consequences for stress recovery dynamics.

Several limitations should be considered when interpreting these findings. First, tissue was collected at 0DPS and 3DPS and behavior was assessed 1–2 days after stress, so the study does not address longer-term persistence of these effects. Second, because our molecular analyses were limited to transcript measurements, the present data cannot determine whether the observed changes in gene expression translate to altered protein abundance, receptor trafficking, or synaptic function within the affected regions. Third, estrous cycle stage was not monitored in adult females, which may have contributed to variability in female-specific outcomes. Additionally, maternal care and litter characteristics were not systematically quantified, so postnatal mediators of the prenatal stress effects observed here cannot be ruled out. Finally, our use of a single repeated restraint paradigm delivered on a fixed schedule was effective in producing a robust corticosterone response, but whether these findings generalize to other stressor modalities or less predictable stress regimens remains to be tested.

In summary, prenatal stress did not broadly amplify canonical physiological or transcriptional responses to repeated restraint stress, as corticosterone, *Fkbp5*, and *Sgk1* were largely unchanged. Instead, its effects emerged as selective, region- and sex-dependent modulation of *Nr3c1* and *Bdnf*, particularly in the hippocampus and prefrontal cortex, consistent with altered stress-responsive plasticity during acute stress and early recovery rather than a generalized baseline deficit. Although physiology was not directly assessed, the regional pattern of transcriptional change is most consistent with preserved hypothalamic stress output but altered hippocampal and prefrontal glucocorticoid- and plasticity-related regulation during stress and early recovery, with selective amygdala inflammatory responsiveness. These findings therefore suggest that prenatal stress primarily biases circuit-level adaptation to later stress rather than broadly amplifying canonical endocrine responses.

## Data Availability

The raw data supporting the conclusions of this article will be made available by the authors, without undue reservation.
