# Supplementary material for: Prenatal stress shapes discrete responses during early recovery from repeated adult stress
Source: Front Cell Neurosci. 2026 May 13;20:1793298. doi: 10.3389/fncel.2026.1793298 (PMC13212063; doi:10.3389/fncel.2026.1793298)
Supplement: Supplementary file 2 [file Table_2.DOCX]

**Supplementary Table 1. TaqMan Assay Primers**

Housekeeping Genes

| **Gene** | **Assay ID** |
| --- | --- |
| *Gapdh* | Mm99999915_g1 |

Genes of Interest

| **Gene** | **Assay ID** |
| --- | --- |
| *Tnf* | Mm00443258_m1 |
| *Il6* | Mm00446190_m1 |
| *Il1b* | Mm00434228_m1 |
| *Nr3c1* | Mm00433832_m1 |
| *Nr3c2* | Mm01241596_m1 |
| *Fkbp5* | Mm00487406_m1 |
| *Crh* | Mm01293920_s1 |
| *Sgk1* | Mm00441380_m1 |
| *Slc2a1* | Mm00441480_m1 |
| *Bdnf* | Mm00432069_m1 |
